# *“You Shall Not Pass”*—tight junctions of the blood brain barrier

**DOI:** 10.3389/fnins.2014.00392

**Published:** 2014-12-03

**Authors:** Hans-Christian Bauer, István A. Krizbai, Hannelore Bauer, Andreas Traweger

**Affiliations:** ^1^Institute of Tendon and Bone Regeneration, Paracelsus Medical University - Spinal Cord Injury and Tissue Regeneration Center SalzburgSalzburg, Austria; ^2^Department of Traumatology and Sports Injuries, Paracelsus Medical UniversitySalzburg, Austria; ^3^Austrian Cluster for Tissue RegenerationVienna, Austria; ^4^Biological Research Centre, Institute of Biophysics, Hungarian Academy of SciencesSzeged, Hungary; ^5^Institute of Life Sciences, Vasile Goldis Western University of AradArad, Romania; ^6^Department of Organismic Biology, University of SalzburgSalzburg, Austria

**Keywords:** blood-brain barrier, tight junctions, PDZ scaffolds, MAGUK proteins, cell polarity, brain capillary endothelial cells, vascular permeability

## Abstract

The structure and function of the barrier layers restricting the free diffusion of substances between the central nervous system (brain and spinal cord) and the systemic circulation is of great medical interest as various pathological conditions often lead to their impairment. Excessive leakage of blood-borne molecules into the parenchyma and the concomitant fluctuations in the microenvironment following a transient breakdown of the blood-brain barrier (BBB) during ischemic/hypoxic conditions or because of an autoimmune disease are detrimental to the physiological functioning of nervous tissue. On the other hand, the treatment of neurological disorders is often hampered as only minimal amounts of therapeutic agents are able to penetrate a fully functional BBB or blood cerebrospinal fluid barrier. An in-depth understanding of the molecular machinery governing the establishment and maintenance of these barriers is necessary to develop rational strategies allowing a controlled delivery of appropriate drugs to the CNS. At the basis of such tissue barriers are intimate cell-cell contacts (*zonulae occludentes*, tight junctions) which are present in all polarized epithelia and endothelia. By creating a paracellular diffusion constraint TJs enable the vectorial transport across cell monolayers. More recent findings indicate that functional barriers are already established during development, protecting the fetal brain. As an understanding of the biogenesis of TJs might reveal the underlying mechanisms of barrier formation during ontogenic development numerous *in vitro* systems have been developed to study the assembly and disassembly of TJs. In addition, monitoring the stage-specific expression of TJ-associated proteins during development has brought much insight into the “developmental tightening” of tissue barriers. Over the last two decades a detailed molecular map of transmembrane and cytoplasmic TJ-proteins has been identified. These proteins not only form a cell-cell adhesion structure, but integrate various signaling pathways, thereby directly or indirectly impacting upon processes such as cell-cell adhesion, cytoskeletal rearrangement, and transcriptional control. This review will provide a brief overview on the establishment of the BBB during embryonic development in mammals and a detailed description of the ultrastructure, biogenesis, and molecular composition of epithelial and endothelial TJs will be given.

## Establishment of the BBB during mammalian embryogenesis

During evolution organisms with a complex nervous system have developed mechanisms to selectively restrict the blood-to-brain traffic of compounds allowing a tight regulation of the neuroparenchymal microenvironment. Such cerebral homeostasis is fundamental for proper function of synapses and neural networks. However, despite the burgeoning literature on when the BBB is established in the course of mammalian development, the topic is still heavily debated. This is largely the result of inconsistent and partially contradictive results stemming from different animal models used and due to misinterpretation of experiments (*for review see Saunders et al. in this volume*). In addition, not a single barrier, but the sum of several cellular and molecular barriers ensures the stable environment needed for the proper function of the central nervous system, further complicating the analysis. Of the three main CNS barriers the brain endothelium constituting the blood-brain barrier (BBB) represents the major interface between the blood and the brain interstitial fluid with an estimated total surface area of 20 m^2^ (Begley and Brightman, 2003). The endothelial cells are fitted with specialized structures, such as cell-cell adhesion complexes obliterating the paracellular space (tight junctions) and an array of transport and shuttle proteins controlling the flux of ions and solutes. The term BBB has been coined nearly 100 years ago by Stern et al. (Stern and Rothlin, 1918; Stern and Gauthier, 1921), implying a structure completely preventing an exchange between blood and nervous tissue. However, since then it has become increasingly clear that the BBB is a complex and dynamic structure allowing a controlled passage of blood-borne substances. In addition, the functional BBB, at least in the adult, is not only composed of the endothelial cells (ECs) lining the microvasculature but also requires the contact and/or input of other cell types, including astrocytes, neurons, and pericytes separated only by a basement membrane. All these contributing building blocks of the BBB are commonly being referred to as “neurovascular unit,” a term which has been introduced for the first time by members of the Stroke Progress Review Group in 2002 at the NIH, USA. During embryonic development however, the composition of the NVU and the nature of the cerebral capillary endothelial cells (cECs) most likely is very different from those seen in the adult. At an even more fundamental level, at which developmental stage can we speak of a structure exerting barrier functions and does BBB formation in cEC coincide with the early vascularization of the growing CNS?

**Figure 1:**
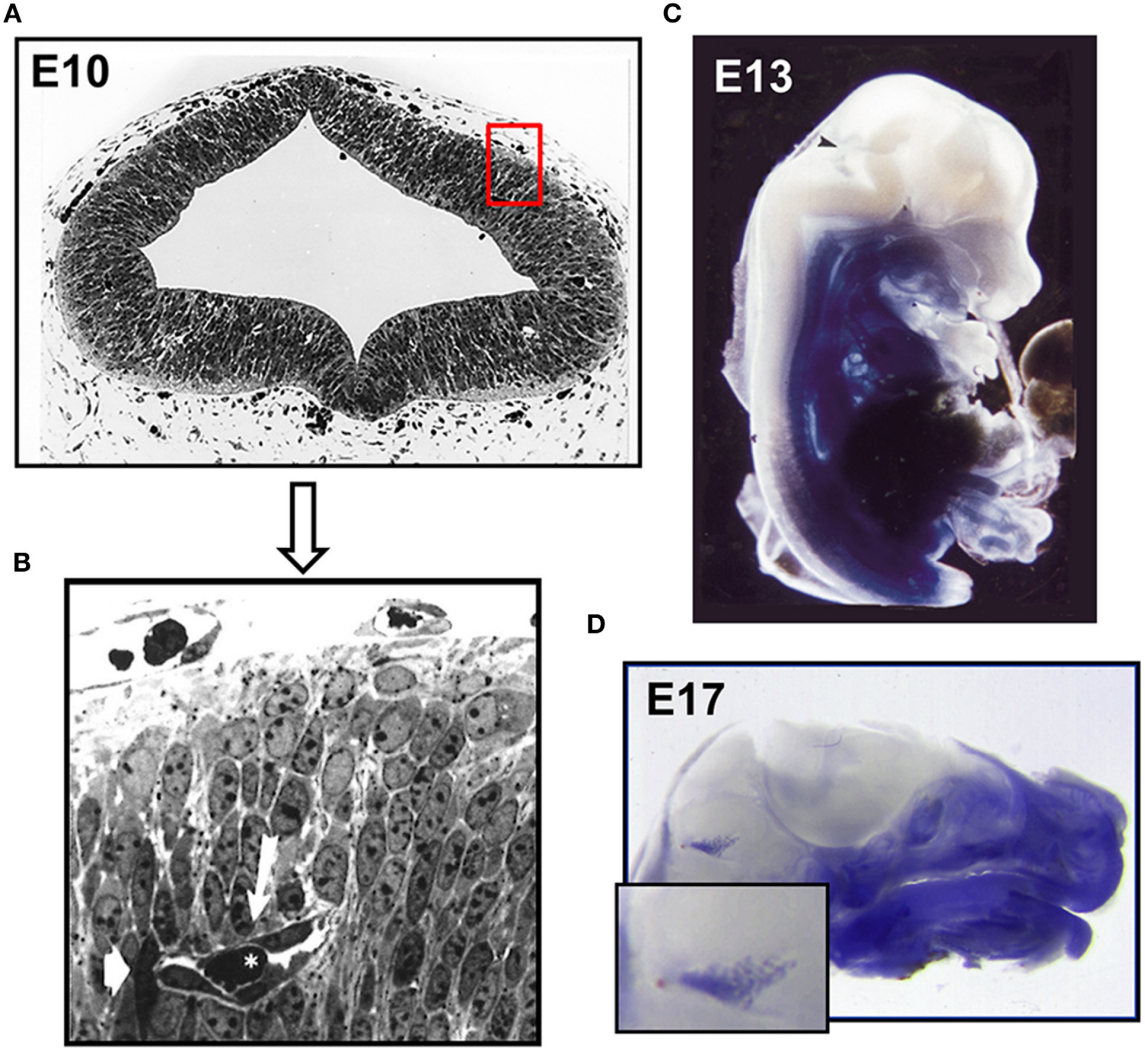
**Establishment of the intraneural vascular plexus and a functional BBB during mouse embryogenesis. (A)** Semithin section of embryonic mouse neocortex at E10 showing the intraneural and parts of the surrounding perineural domain **(B)** neuroepithelial cells surround a primitive capillary (white arrow) containing a red blood cell (asterisk). Murine E13 **(C)** and E17 **(D)** embryos microperfused with 0.5% Trypan blue solution. After fixation specimens were cut medially and photographed. Arrowhead indicates and enlarged area depicts stained choroid plexus (Bauer et al., 1993, 1995).

In mice angiogenesis in the brain begins at day E9 of development, when pial vessels encircle the telencephalon (Vasudevan et al., 2008). By E10 pial vessels sprout and start forming a primitive vascular plexus within the telencephalon. The newly formed vessels have a premature appearance (see Figures 1A,B) but allow already erythrocyte traffic (Bauer et al., 1993, 1995). In parallel, periventricular vessels migrate into the dorsal telencephalon resulting in a ventrolateral to dorsomedial angiogenesis gradient (Vasudevan et al., 2008). It is proposed that the pial vessels develop into venous sinuses and the periventricular vessels into arteries. The assumption that the plexus of the CNS likely arises from two different endothelial cell populations is further supported by the demonstration of different expression patterns of homeobox transcription factors, namely Dlx1/2, Nkx2.1 in periventricular ventral vessels and Pax6 in pial vessels. Intriguingly, these transcription factors also regulate neuroepithelial cell proliferation and neuronal migration (Sussel et al., 1999; Nery et al., 2003). Therefore, it is tempting to speculate that angiogenesis in due course influences and regulates neuronal proliferation and differentiation.

Several proteins, morphogenetic molecules and their receptors are involved in both, angiogenesis of the brain and in the formation of the BBB: VEGF has been identified as one of the first of these effectors. This morphogenetic molecule is secreted by neuronal precursor cells and promotes via binding to endothelial tyrosine kinase receptors (FLT-1/VEGFR-1; FLK-1/VEGFR-2) vessel sprouting during the early phases of CNS development (Breier et al., 1992; Raab et al., 2004). VEGF is also secreted by brain endothelial cells and exerts its influence on the cortical cytoarchitecture during later stages of embryonic development allowing the establishment of a functional neuronal architecture in the CNS (Li et al., 2013). Other effector molecules include members of the Wnt family (Wnt7a/b, Wnt3a) together with the action of ß-catenin, Tie-2 (an endothelial cell-specific receptor tyrosine kinase) and its ligands angiopoietin-1 and angiopoietin-2 (Ang-1, Ang-2), as well as sonic hedgehog and Norrin (Engelhardt and Liebner, 2014). The important role of Ang-1 during BBB formation is additionally strengthened by the identification of SSeCKS, a soluble protein which induces the expression of Ang-1 in cEC of the BBB (Lee et al., 2003). Next to these effectors which exert their action throughout the entire CNS, others are restricted to specific areas. For example GPR124 (orphan G protein-coupled receptor 124) is specifically expressed in endothelial cells regulating sprouting in the forebrain and in spinal cord but not in other regions of the CNS (Kuhnert et al., 2010). GPR124 also modulates TGF-ß signaling, thereby influencing pericyte and astrocyte differentiation and further impacts upon glucose transport by regulating the expression of Glut-1 in cECs. Taken together, the factors described above are intimately related to the induction of a functional BBB as they promote differentiation of the cECs, ultimately resulting in the formation of occluding cell adhesion structures and the expression of specific transporter receptors and shuttling molecules such as the multidrug resistance molecule.

The early transplantation experiments by Stewart and Wiley and Ikeda et al. demonstrated the induction of BBB features of invading endothelial cells by ectopic transplantation of embryonic brain tissue, indicating that the acquisition of BBB function is not merely an intrinsic property of cerebral endothelial cells (Stewart and Wiley, 1981; Ikeda et al., 1996) but requires the direct and indirect communication with neighboring neuronal cells. In the early brain these are the neuroepithelial cells and neuroblasts and later in embryonic development and postnatally, neurons, pericytes and glial cells (Bauer and Bauer, 2000). However, the underlying molecular machinery and ramifications of this interaction are not fully understood mainly due to its complex nature and its constant modulation during embryonic and postnatal development. Lindahl et al. showed that platelet derived growth factor–B (PDGF-B) secreted from migrating cECs led to the recruitment of pericytes and their attachment to the newly formed vessels (Lindahl et al., 1997). In the following years *in vivo* and *in vitro* studies demonstrated that pericytes are imperative for BBB formation (Deli et al., 2005; Armulik et al., 2010; Daneman et al., 2010b). Recently, another effector was described by Ben-Zvi et al. (2014) named Mfsd2a, a transmembrane protein exclusively expressed in cerebral endothelial cells. Mfsd2a suppresses transcytosis in endothelial cells and thus the transport of plasma proteins. Interestingly, the protein is expressed in mice at embryonic day 13.5 and its expression depends on the presence of pericytes. In Mfsd2a-knockout mice the BBB is impaired from embryonic day 15.5 to adulthood what led the authors to the conclusion that vascularization and the establishment of a functional BBB are not coinciding. In earlier findings however it has been shown that the BBB becomes functional even before E15 (Bauer et al., 1993, 1995). Microperfusion of E12–E13 mouse embryos with Trypan Blue revealed that the CNS remained void of staining except for the choroid plexus anlage, indicating that at least certain plasma proteins have been excluded (see Figures 1C,D). Further, the number of pinocytotic vesicles in endothelial cells of intraneural capillaries decreased from E12 onwards, whereas the number of vesicles in endothelial cells of the perineural domain in the CNS increased (see Table 1).

**Table 1:** **Frequency of endothelial fenestrations (f), junctional complexes (j.c.) and pinocytotic vesicles (p.v.) in intraneural and perineural capillaries during murine embryonic development**.

**Day of embryonic development**	**Intraneural capillaries**			**Perineural capillaries**		
	**f**	**j.c.**	**p.v.**	**f**	**j.c.**	**p.v.**
E9		No capillaries present		0.9	3.2	2.0
E10	nd	1.7	2.9	0.13	2.9	1.9
E12	nd	2.58	2.33	nd	3.25	3.0
E14	nd	2.29	1.57	nd	2.43	3.29
E17	nd	2.25	1.63	nd	2.75	4.13

*Values were determined as follows: number of f, j.c., or p.v. divided by the number of capillaries examined (7–17 capillaries; mean values are shown). n.d., not detected (Bauer et al., 1993)*.

In summary, the formation of a functional BBB, or more precisely, of a neurovascular unit with specific barrier structures and transport functions is a complex, multifactorial process requiring the concerted action and interaction of various cell types including cECs, astrocytes, neuronal and glial cells, as well as pericytes. Finally, although the developmental stage at which a functional BBB is present is still under debate, there is good evidence that a barrier toward large molecules exists very early in embryonic brain development, which then fully matures postnatally.

## Tight junction morphology and function

Next to an extensive transport machinery, tight junctions sealing the microvascular endothelium represent the core structure of the BBB. The tight junction (TJ) is the most apical cell-cell junction complex in polarized epithelia and endothelia and can be visualized by ultrathin-section electron microscopy as focal points or “kissing points” where membranes of adjacent cells come into close apposition, completely obliterating the intercellular cleft (Farquhar and Palade, 1963). The application of freeze-fracture electron microscopy provided a more detailed description of TJs. TJs of epithelial cells appear as a complex network of anastomosing and continuous cylindrical strands on the protoplasmic leaflet (P-face) of the plasma membrane, with complementary grooves on the exoplasmic leaflet (E-face), completely encircling the apical aspect of the cell (Chalcroft and Bullivant, 1970; Staehelin, 1973, 1974; Van Deurs and Koehler, 1979). In contrast, depending on the vascular bed investigated, TJ strands of endothelial cells generally display a significantly lower level of P-face association. Whereas endothelial cells of the peripheral vasculature show predominantly E-face associated strands (Simionescu et al., 1976; Muhleisen et al., 1989), freeze fracture replicas of TJs located at the BBB reveal a high degree of P-face association and are among the most complex found in the entire vasculature (Nagy et al., 1984; Liebner et al., 2000b). It seems that not only the complexity of the strand network but also the association of the TJ-strands with the P- or E-face reflects the functional quality (i.e., permeability and electrical resistance) of the barrier (Lippoldt et al., 2000). The varying P-face/E-face ratios possibly are a consequence of altered attachment of the TJ strands to the epithelial or endothelial cytoskeleton, but much detail is still missing.

Next to the establishment and maintenance of a size and charge selective paracellular barrier (Gumbiner, 1987, 1993; Cereijido et al., 1993), TJs also serve to create an intramembrane diffusion barrier (“fence”) restricting the intermixing of apical and basolateral components within the exoplasmic leaflet of plasma membranes (Van Meer et al., 1986). In addition, they guide a selective distribution and clustering of membrane components to distinct cell-surfaces (Fanning and Anderson, 1999) and assemble large protein complexes around scaffolding proteins at their cytoplasmic aspect. The modular nature of these adaptor proteins allows the spatial and temporal assembly of multi-protein complexes not only ensuring the structural integrity of the TJ, but also integrating various regulatory pathways which are pivotal for TJ physiology, i.e., establishment of apico-basal polarity, gene expression, and cell proliferation (Gonzalez-Mariscal et al., 2008; Guillemot et al., 2008; Balda and Matter, 2009; McCrea et al., 2009). Finally, the junctional cytoplasmic plaque provides a direct and indirect link to the cytoskeleton.

Generally, much of our understanding of TJ biology stems from studying polarized epithelial cells. However, several aspects of the core structure of endothelial and epithelial TJs are similar and many of the reported findings apply most likely for both cell types. The identification of an ever increasing number of TJ-associated proteins (Gonzalez-Mariscal and Nava, 2005; Wang and Margolis, 2007; Ebnet, 2008; Gonzalez-Mariscal et al., 2008; Giepmans and Van Ijzendoorn, 2009; Steed et al., 2010; Bauer et al., 2011) has led to a wide acceptance of the “protein model”, proposing that linear arrays of polymerized transmembrane adhesion molecules form TJ-strands and adhere with strands from apposing plasma membranes (Staehelin, 1973; Tsukita et al., 2001; Furuse, 2010). However, it remains to be unequivocally proven that the high transcellular electrical resistance of epithelia and endothelia can solely be attributed to the molecular interaction of TJ-strands. The involvement of lipid components (“lipid model”) cannot be fully ruled out and a “lipid-protein hybrid model” might explain the complexity of TJ physiology (Kan, 1993; Yamagata et al., 2003; Lee et al., 2008; Chen-Quay et al., 2009). But information on the functional and structural relationship of proteins and lipids in the junctional complex remain scarce.

## The biogenesis of tight junctions

Functional TJs not only seal the paracellular space between epithelial and endothelial cells, they are also imperative for the establishment of a polarized, transporting epithelial and endothelial phenotype. The cellular constituents of tissue barriers are polarized, displaying apico-basal polarity through the selective distribution of specific lipid- and protein complexes to distinct sites at the cell surface. Generally, the cells are oriented with their apical aspect of the plasma membrane toward the lumen (“free surface”), whereas the lateral domains are in close contact with neighboring cells and basally adhere to other solid tissues (or parenchyme), only separated by a basement membrane. Epithelial and endothelial cells grown in suspension, or loss of matrix anchorage results in programmed cell death (anoikis), whereas grown on a surface, even single cells polarize. Therefore, the contact to the extracellular matrix, mainly conferred by the family of integrin transmembrane proteins, is crucial to ensure robust cell polarization and morphogenesis *in vivo* (Lee and Streuli, 2014). As soon as an appropriate vectorial transport machinery is in place, epithelia and endothelia are considered fully differentiated, separating tissue and organ compartments and allowing the establishment and maintenance of distinct internal and external milieus.

However, in spite of considerable structural similarities the functional properties of epithelial and endothelial barriers in various tissues and organs can significantly vary. This is largely due to the expression of different transmembrane (i.e., claudins; see further below) and accessory TJ proteins. However, also parameters other than mere structural features need to be taken into account when comparing barriers of various origin and organs. For example, environmental factors (i.e., soluble factors or contacting cells) can influence the barrier properties in developing and mature organs. A beautiful example is the intimate association of cerebral endothelial cells with neuroectoderm-derived accessory cells (astroglia, neurons, and neural precursor cells), which is a unique feature of the BBB and imperative for its physiological functioning. These cells not only contribute physically to the barrier but also induce barrier properties in differentiating cerebral endothelial cells (Bauer and Bauer, 2000; Abbott, 2002; Haseloff et al., 2005; Abbott et al., 2006; Weidenfeller et al., 2007).

Most of our knowledge about tissue barrier formation stems from studies on epithelial and endothelial cells *in vitro* (Gonzalez-Mariscal et al., 1985; Stevenson et al., 1988; Bauer et al., 2011; Watson et al., 2013; Czupalla et al., 2014; Wilhelm and Krizbai, 2014). In this respect, particularly the re-establishment of TJs following calcium depletion and the concomitant breakdown of cell-cell contacts has been investigated (Gonzalez-Mariscal et al., 1990; Contreras et al., 1992; Stuart et al., 1994). Much of our understanding of TJ formation in primary (and secondary) epithelia also comes from studying embryogenesis of *D. melanogaster* (cellularization) and *C. elegans* (Knust and Bossinger, 2002; St Johnston and Ahringer, 2010). Mammalian TJ biogenesis has mainly been described in cultured preimplantation mouse embryos, which serve as a valuable model to determine the expression of junctional proteins during the earliest steps of cell lineage differentiation. Cultured preimplantation mouse embryos can easily be manipulated to precisely determine the role of junction-related proteins at early developmental stages such as compaction and cavitation (formation of the blastocoel) (Dard et al., 2008; Eckert and Fleming, 2008). Early signs of junction establishment appear during formation of tubular structures or cavities, a process which is fundamental to organ development in both vertebrates and invertebrates. From a range of investigations describing the epithelial and endothelial tubular biogenesis, three major morphologic mechanisms are being proposed, commonly referred to as *cell hollowing*, *cord hollowing*, and *cavitation*. In all of them, tubes or cavities arise from lumenless, unpolarized cell aggregates or single unpolarized cells. While cell and cord hollowing are common in vascular structures, cavitation is rather attributed to epithelial tubulogenesis and is only scarcely observed in endothelial vessel formation (Lubarsky and Krasnow, 2003). Cavitation has extensively been described in the preimplantation mouse embryo and coincides with the first occurrence of a differentiated epithelium in the mammalian embryo (Eckert and Fleming, 2008). The undifferentiated mammalian blastomeres are subjected to three rounds of cleavage, resulting in the formation of the eight-cell embryo, also referred to as the “early morula” stage. Subsequently, compaction of the embryo is initiated, representing the moment of establishment of an epithelial phenotype and the generation of an apical-basal axis. This asymmetric cellular organization is then coupled with a specific orientation of the mitotic spindle during cell division, resulting in asymmetric division of the blastomeres giving rise to two different cell populations. The inner, non-polarized blastomeres form the inner cell mass (ICM) of the blastocyst, and the outer polarized blastomeres giving rise to the first polarized epithelium, the trophectoderm (TE) which develops into the chorioallantoic placenta later during development. The ICM then develops into the epiblast, the origin of all tissues of the embryo proper, and the primitive endoderm which gives rise to the extra-embryonic membranes. A more detailed description on embryonic axis establishment and blastocyst lineage formation is beyond the scope of this review and the reader is referred to a series of excellent reviews (Larue et al., 1994; Arnold and Robertson, 2009; Rossant and Tam, 2009; Morris and Zernicka-Goetz, 2012; Takaoka and Hamada, 2012).

Overall, premature intercellular contacts serve as an initial spatial cue for the establishment of polarity not only in blastomeres but also in cells in general. Early studies then identified E-cadherin as the prime epithelial signature marker. During compaction of the early mouse embryo, diffusely localized E-cadherin becomes redistributed to long filopodia protruding from the blastomeres which facilitate the attachment of two neighboring cells via homophilic binding of opposing E-cadherin clusters (Fierro-Gonzalez et al., 2013). Interestingly, mouse embryos deficient in E-cadherin fail to form a proper trophectoderm and die around the time of implantation, compaction not being affected. This late phenotype is explained by the presence of a residual maternal E-cadherin pool, which is sufficient to drive compaction (Larue et al., 1994). Next to E-cadherin, the homophilic binding of nectin family members at developing adhesive structures takes place very early during the establishment of the polarized epithelial phenotype (Sato et al., 2006; Takai et al., 2008b). Nectins constitute a family of single-pass, calcium-independent transmembrane adhesive molecules which, together with clusters of cadherins form mature adherens junctions (Indra et al., 2013). During the early phases of polarization it is believed that nectin controls the initiation of cadherin clustering by preventing the endocytotic removal of cadherin (Takai et al., 2008a). The majority of nectin function is conferred via its association with the cytoplasmic protein afadin, which allows cross-talk with other junctional complexes. Afadin not only functionally couples the E-cadherin and nectin complex (Tachibana et al., 2000), but also directly interacts with the TJ-associated protein JAM-A, thereby influencing tight junction assembly during further progression of the polarity program (Fukuhara et al., 2002). JAM-A assembles at the cell membranes of the early 8-cell mouse embryo prior to compaction and unlike any other TJ constituent its recruitment to the cell-cell contact initiation sites is independent of cadherin-mediated adhesion (Thomas et al., 2004). Therefore, JAM-A recruitment occurs substantially ahead of other TJ transmembrane proteins, with occludin and claudin assembly taking place from the late morula stage onwards (Thomas et al., 2004; Eckert and Fleming, 2008). Importantly, only compact embryos treated with an E-cadherin neutralizing antibody disassemble rapidly, whereas inactivation of JAM-A does not perturb compaction and only results in a delayed cavitation of the embryo (Thomas et al., 2004). This result underscores the importance of cadherin-based cell-cell adhesion during compaction and indicates that JAM-A cell surface expression does not contribute to the initiation of cell adhesion *per se* within the embryo.

## Cell polarization—a key step toward barrier formation

The establishment of mutually exclusive cortical domains in epithelia and endothelia in part relies on the asymmetric distribution of a set of evolutionary conserved biochemical factors controlling cell polarity. Genetic studies in *C. elegans* and *D. melanogaster* have identified three polarity groups, the Crb, Par, and Scrib groups, with the members of each group having interdependent and partly antagonistic functions. Although the majority of our understanding of the complex polarity signaling pathways in mammals is based on studies carried out in 2D and 3D cultured epithelial cells more recent studies indicate that the same set of proteins is critical for endothelial polarity (Lizama and Zovein, 2013). The Par complex is located sub-apically in epithelial cells and assembles around Par-6 isoforms (Macara, 2004). Par6 binds atypical protein kinase C (aPKC) λ/ι or ξ and selectively engages GTP-bound Cdc42 (Joberty et al., 2000; Johansson et al., 2000; Lin et al., 2000), which stimulates the activity of Par6-associated aPKC several-fold, potentially through a conformational rearrangement of the complex. The Par6/aPKC complex appears to be a general determinant of polarity, which undergoes cell-specific interactions with other polarity proteins. Members of the Par-complex act in synergy with the Crumbs/Pals/Patj group of proteins to define the apical cell surface (Hurd et al., 2003; Ebnet et al., 2008). As Jam-A (and all other isoforms; see further below) directly interacts with Par3, it most likely serves as a spatial landmark to tether the Par3/Par6/aPKC polarity complex to the primordial junction. The expression of the Par6/aPKC complex has been confirmed in brain endothelial cells (Daneman et al., 2010a) and it has also been shown to be important for the establishment of endothelial polarity and lumen formation in general (Zovein et al., 2010). Genetic deletion of β 1-integrin results in decreased Par3 expression, leading to a loss of endothelial polarity (Osada et al., 2011). More recently, it has been shown that the non-canonical Wnt/planar cell polarity (PCP) pathway contributes to TJ integrity of hCMEC/D3 cell *in vitro*, suggesting that this pathway might act as a key regulator of the BBB *in vivo* (Artus et al., 2014). However, similarly to what has been reported for epithelial cells (Chen and Macara, 2005; Qin et al., 2005) the knock-down of Par3 only delays but does not fully inhibit TJ formation in brain endothelial cells.

The Crumbs group (Crb) localizes apically or at apical junctions and consists of the transmembrane Crb proteins and the large scaffolding proteins Pals1 and Patj through which the complex physically is connected to the Par group via Par6 (Bilder et al., 2003; Hurd et al., 2003; Assemat et al., 2008; Tepass, 2012). In ECs tight junction integrity is also maintained by angiomotin and angiomotin-like proteins (Amot, AmotL1), in part through the action of the associated RhoGEF Syx and loss of any of these proteins results in increased vascular permeability and/or reduced sprouting angiogenesis (Garnaas et al., 2008; Zheng et al., 2009; Ngok et al., 2012).

The basolateral region of epithelial cells is specified by a group of tumor suppressor genes, the Scrib complex. These include Scribble, which encodes a LAP protein (with leucine-rich repeats and four PDZ domains), Dlg, a MAGUK protein, and Lgl, comprised of WD40 repeats (Yamanaka and Ohno, 2008). Scrib, Dlg and Lgl are membrane-associated adaptors implicated in cytoskeletal organization and protein trafficking and potentially in coupling tissue architecture to withdrawal from the cell cycle. Although little is known about Scribble in endothelial cells, a recent study provides evidence that loss of Scrib does exhibit vascular hemorrhaging suggestive of defective permeability barriers and a likely role in endothelial cell tube formation and angiogenesis (Michaelis et al., 2013). In breast cancer epithelia Scribble has been demonstrated to interact with TAZ, a member of the Hippo signaling pathway (Cordenonsi et al., 2011). Interestingly, knock-down of angiomotin-like 2 (amotL2), another member of the hippo cascade with ties to the Crb-polarity complex, results in decreased tubulogenesis *in vitro* (Wang et al., 2011). Therefore, it is tempting to speculate that the opposing functions of Crumbs/Amot and Scribble polarity complexes also antagonistically impact upon the Hippo pathway to regulate endothelial polarity and lumen formation.

One of the essential polarity regulators from yeast to man is the RhoGTPase Cdc42. In epithelial cells PTEN-mediated segregation of phosphoinositides has been shown to initiate polarization by recruiting Cdc42 to apical domains (Martin-Belmonte et al., 2007). Further, the small GTPase Rac1 has been shown to play an important role downstream of the Par complex (Iden and Collard, 2008). Independent of their polarity actions the best studied function of Rho GTPases is their role als molecular organizers of the actin cytoskeleton. These two processes are interconnected as cell polarization and the establishment of a functional TJ also entails a complex rearrangement of the actin cytoskeleton, microtubule organizing centers, and the establishment of a vesicle trafficking machinery, all together facilitating vectorial transport functions (Rodriguez-Boulan and Macara, 2014).

After establishment of cell polarity additional junctional proteins are being sequentially delivered to the site of the adhesion structure resulting in the maturation of the adherens junction (AJ) and TJ (Eckert and Fleming, 2008). Initially, during compaction an immature junction, containing AJ and TJ constituents, is formed serving as an initial landmark which is required for proper cell polarization. Next to cadherin-catenin components, the TJ-related proteins ZO-1 (the shorter ZO-1α-isoform) and JAM-A localize to the immature junction. In addition, the rab GTPase rab13 relocates early to the apico-lateral contact site (Sheth et al., 2000). During the following 16-cell stage the TJ proteins ZO-2 (Sheth et al., 2008) and cingulin (Javed et al., 1993) enter the stage of junctional biogenesis and subsequently the transmembrane proteins occludin and claudin-1 and -3 are targeted to the junction. Concomitantly the segregation into an apical tight junction and an adherens junction located more basally takes place (Rajasekaran et al., 1996; Sheth et al., 2000). Interestingly, although the formation of tight junctions and cell polarity are intimately related, *in vitro* the Par protein- and Scribble-complex have minor effects on TJ establishment in epithelial cells. The knock-down of either protein only delays but does not inhibit TJ formation (Chen and Macara, 2005; Qin et al., 2005). In contrast, perturbation of the Crbs/Pals1/Patj complex results in persistent TJ defects (Fogg et al., 2005; Shin et al., 2005; Wang et al., 2007). As the junction further matures, together with a complex rearrangement of the cytoskeleton and the establishment of a vectorial transport system, a permeability seal is formed which is the prerequisite for the formation of the nascent blastocoel cavity.

Similar to epithelial differentiation, blood vessel formation relies on cell-cell contact and apical-basal polarization of endothelial cells prior to lumen formation and junctional maturation. As already mentioned above, various models have been proposed to explain vascular lumenization, including the intracellular vacuole coalescence model (*cell hollowing*), or extracellular vacuole exocytosis and lumenal repulsion model (*cord hollowing*) (Iruela-Arispe and Davis, 2009; Zeeb et al., 2010; Axnick and Lammert, 2012). While cell hollowing involves the generation and fusion of intracellular vacuoles of individual cells, cord hollowing is based on lumen formation between two or more ECs (or an EC cord) (Lubarsky and Krasnow, 2003; Blum et al., 2008; Chung and Andrew, 2008; Lee and Bautch, 2011). Both types of lumen formation make use of a similar set of proteins and pathways and distinguishing them from each other *in vivo* is a difficult task. Generally, lumina of small capillaries (maximum diameter of a single cell) are formed by cell hollowing while lumenization of larger vessels follows the process of cord hollowing. However, using a computational model of lumen formation it was recently suggested that cell hollowing and cord hollowing may operate in parallel (Boas and Merks, 2014).

**Figure 2:**
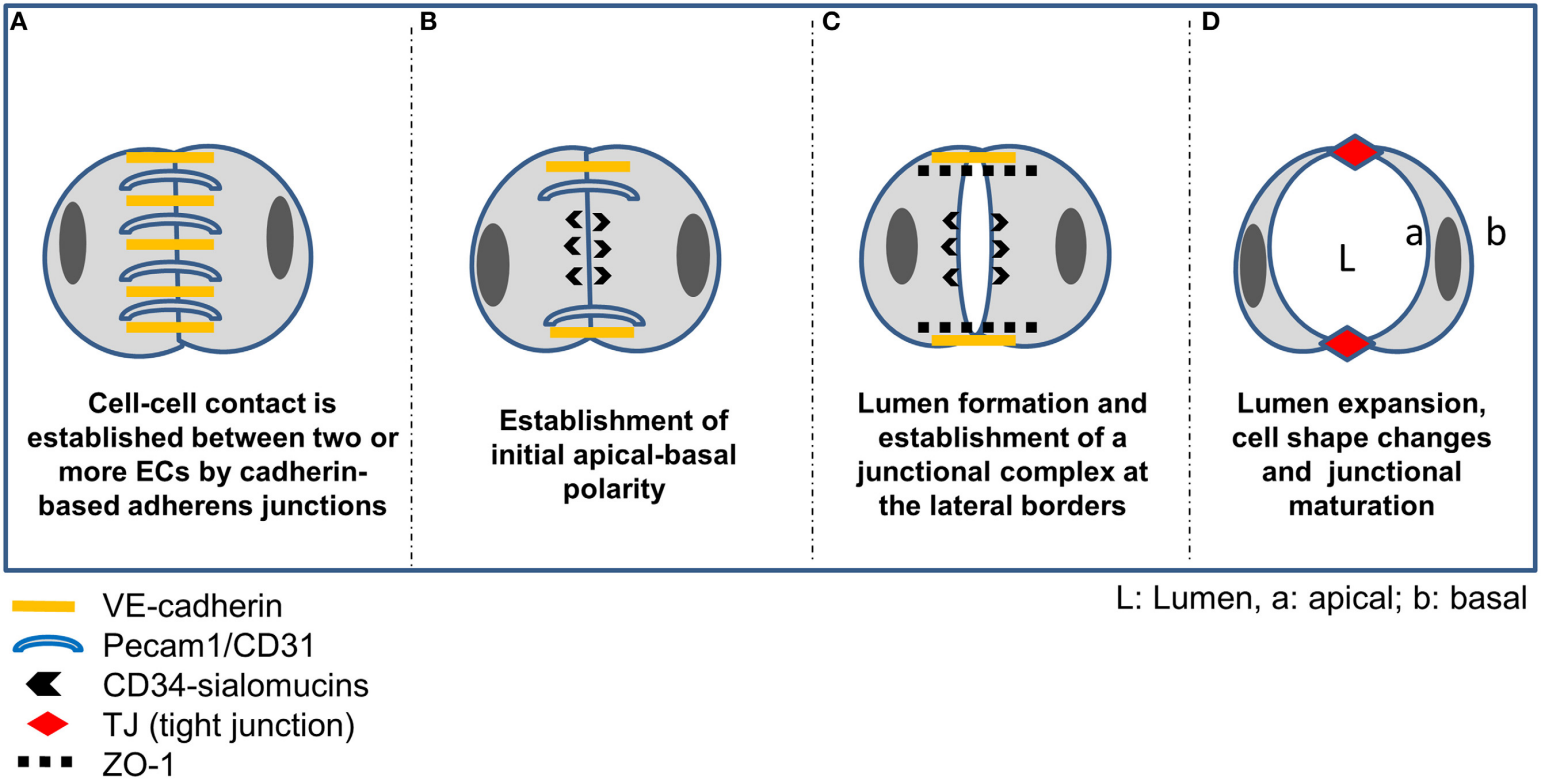
**Vascular lumen formation by cord hollowing. (A)** Intercellular adhesion of two or more ECs is accomplished by homophilic interaction of VE-cadherin and Pecam-1. **(B)** Following establishment of Par3-mediated apicobasal polarity via ß-1 integrin matrix interaction and the adaptor protein RASIP1, CD34-sialomucins (CD34 and podocalyxin; PODXL) are recruited to cell-cell contacts delineating the future apical border. VE-cadherin is required for specifically targeting CD34-sialomucins to contact sites, and the delivery depends on PTEN-mediated transformation of PIP3 to PIP2. Rasip1 localizes at inter-endothelial junctions and acts downstream of Rap1 (Ras-related protein1), mediating EC-ECM adhesion and stabilizing junctional integrity by actin bundling. In cooperation with Radil (ras-association and dilute domain-containing protein), Rasip1 further inhibits Rho-mediated stress fiber formation, thereby increasing endothelial barrier function. Further, CCM1 mediates the stabilization of β-catenin-containing endothelial cell-cell junctions downstream of the Rap1 GTPase. **(C)** Due to the negatively charged CD34-sialomucins, electrostatic repulsion leads to separation of the contacting plasma membranes thereby creating a free (non-contacting) surface and a small lumen between ECs. Concomitantly, cadherin-based AJs are redistributed to lateral borders, indicating the establishment of a lateral junctional complex. Following phosphorylation by protein kinase C, pMoesin, the major ERM protein in endothelial cells, is recruited to the future apical membrane and links CD34-sialomucins to F-actin, supporting cytoskeletal rearrangements in ECs during lumen expansion. Further, non-muscle myosin assembles at the apical plasma membrane and interacts with F-actin in response to VEGF signaling to support lumen expansion and cell shape changes. **(D)** Further maturation of the endothelial TJs is then mediated by claudin-5, occludin, members of the junctional adhesion molecule (JAM) family, or by EC-selective adhesion molecule (ESAM).

The initial phase of cord hollowing is characterized by cell-cell adhesion, exocytosis and apical membrane repulsion (see Figure 2). Initial intercellular contacts are established by homotypic interaction of VE-cadherin and Pecam-1/CD31 which are distributed along the entire contacting plasma membrane. Additionally, the uniform distribution of the polarity proteins Par3 in contacting ECs is indicative of a yet unpolarized state of the cells. Besides VE-cadherin also N-cadherin was found at EC contact sites, which is believed to increase the amount of VE-cadherin at cell-cell contacts (Luo and Radice, 2005). Subsequently, de-adhesive glycoproteins (CD34-sialomucins) are delivered from cytoplasmic vesicles to the contacting plasma membrane delineating the future apical surface. The specific targeting of CD34-sialomucins to EC contact sites depends on VE-cadherin and involves PTEN signaling together with PIP2/PIP3 conversion (Zeeb et al., 2010). It is believed that the negatively charged extracellular domains of CD34 sialomucins initiate the repulsion of opposing leaflets creating a narrow space between the cells, commonly referred to as “slit formation” (Robbins and Beitel, 2010; Strilic et al., 2010). One of the CD34-related sialomucins is podocalyxin (PODXL). Originally, PODXL has been described as the major sialoprotein in the glycocalyx of glomerular podocytes (Kerjaschki et al., 1986). However, it has been recognized early that PODXL is a widespread component of vascular endothelial cells (Born and Palinski, 1985; Horvat et al., 1986). It localizes to the luminal domain of endothelial plasma membranes in a patchy distribution and is absent from the abluminal aspect (Horvat et al., 1986). Although the function of endothelial sialoproteins has long remained elusive, a role in regulating the thrombogenic properties of the endothelial surface was proposed (Gorog et al., 1982; Born and Palinski, 1985). More recently it has been shown that PODXL (syn. gp135) is a key regulator of apical domain structure establishment in epithelial cells (Meder et al., 2005) and it recruits filamentous actin and ezrin to the plasma membrane, inducing microvillus formation (Nielsen et al., 2007). Interestingly, embryoid bodies lacking ZO-1 or ZO-1/ZO-2 (but not ZO-2 alone) exhibit reduced expression of PODXL at the apical plasma membrane and lack TJs in the extraembryonic endoderm (Phua et al., 2014). Further, quantitative PCR profiling of RNA samples from laser capture microdissected microvessels revealed that PODXL is preferably expressed in brain microvessels (Agarwal et al., 2010) suggesting a role in BBB establishment and/or function.

CD34-sialomucins are further linked to the actin cytoskeleton via protein kinase C- (PKC) phosphorylated moesin, thereby contributing to cell shape changes during lumenization (Strilic et al., 2009). Lumen extension then depends on cytoskeletal rearrangements involving non-muscle myosin and recruitment thereof to the apical F-actin is accomplished by activation of the Rho-associated protein kinase (ROCK) and VEGF-A signaling (reviewed in Lee and Bautch, 2011). Finally, the assembly of CD34-sialomucins at the future apical membrane triggers the redistribution of junctional proteins to the lateral border, constituting the initial step in the formation of a lateral junctional complex.

**Figure 3:**
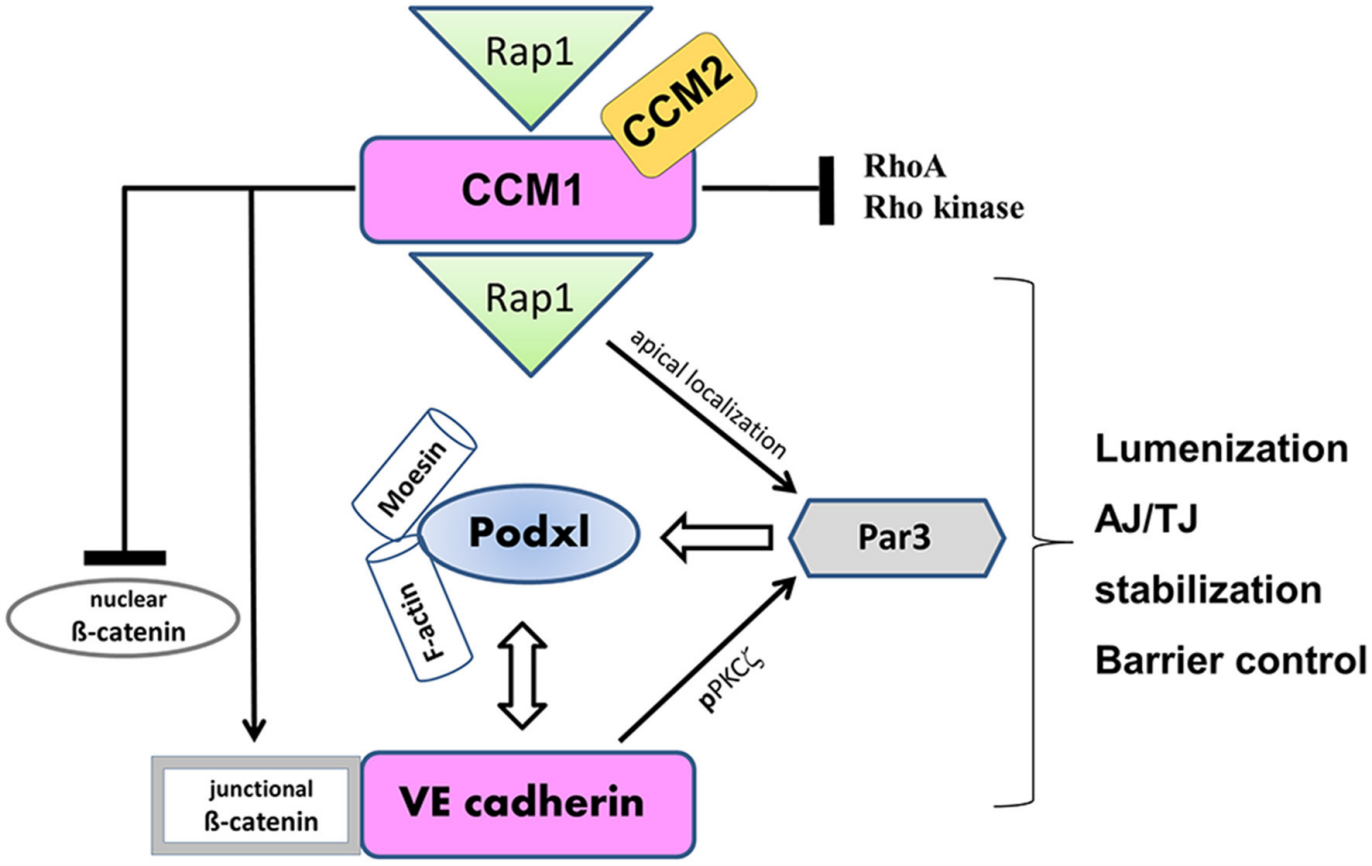
**VE-cadherin and CCM1 act in concert to establish endothelial polarization and lumen formation**. Rap1 regulates the junctional localization of CCM1 and mediates CCM1 activity in endothelial polarization and lumen formation. CCM1 stabilizes VE-cadherin at AJs, by associating with ß-catenin and inhibiting dissociation of ß-catenin from AJs. Thereby, nuclear accumulation of ß-catenin and ß-catenin transcriptional signaling is impaired. VE-cadherin promotes the apical localization and activation of the Par3 polarity complex. While VE-cadherin is needed for phosphorylation of PKCξ, CCM1/Rap1 is responsible for the junctional recruitment of the polarity complex. The accumulation of podocalyxin (PODXL) at the presumptive apical membrane is controlled by VE-cadherin and ß1 integrin. PODXL in turn recruits moesin and filamentous actin (F-actin) to the apical domain initiating endothelial lumen formation.

Disturbance of endothelial polarization is observed in cerebral cavernous malformations (CCM), the most common vascular dysplasia in the brain, which is characterized by hemorrhagic stroke, focal neurological deficits and seizures (reviewed in Draheim et al., 2014). CCM1 binds to and is regulated by the small GTPase Rap1, enhancing the adhesive properties of VE-cadherin (Glading et al., 2007). The CCM1 FERM domain is unmasked by the activated Rap1, targeting CCM1 to the junction and promoting endothelial polarization in a Par-dependent manner (Lampugnani et al., 2010) (see Figure 3). In the absence of CCM1, endothelial lumenization is severely disturbed, as evidenced by grossly dilated capillaries and multicavernous structures (Clatterbuck et al., 2001). Endothelial-specific disruption of the CCM1 gene in mice induces endothelial to mesenchymal transition due to increased BMP6–SMAD signaling, a process which is characterized by the loss of cell polarity, increased cell proliferation and migratory capacity (Maddaluno et al., 2013). Although CCM genes are inactivated in all types of endothelial cells under pathological conditions, CCM lesions are mostly apparent in the brain vasculature. Therefore, the loss of endothelial polarity and the concomitant impaired astroglia-endothelial interaction, which is imperative for BBB function, seems to be the underlying cause of frequent vascular lesions in the brain (Maddaluno et al., 2013). This and other studies elegantly demonstrate that cell-cell adhesion and apical-basal polarization appears to be a universal event preceding epithelial and endothelial tubulogenesis, a mechanism imperative for organ development and vascularization.

Results from numerous studies on various developmental models converge on a crucial role for apical membrane specialization prior to junctional maturation. The establishment of a cadherin-based primordial junctional complex prior to segregation of a more apically located TJ appears to be a conserved step in epithelial and endothelial differentiation. Besides the indispensability of cadherin-based cell-cell contacts, the early and bi-phasic role of ZO-1 isoforms appears to be crucial for endothelial and epithelial barrier formation. Following *de novo* establishment of tight junctions, a dizzying number of proteins localize to the cytoplasmic aspect of TJ not only ensuring the integrity of the barrier but also allowing a remodeling and change in permeability properties in response to physiological and pathological stimuli. Of the known TJ constituents, the *zonula occludens* (ZO) proteins lie at the heart of the TJ cytoplasmic plaque. Below, the major tight junction proteins are being discussed, focusing on proteins that have been shown to localize to endothelial TJs.

## Tight junction proteins

### Membrane-spanning TJ constituents

**Figure 4:**
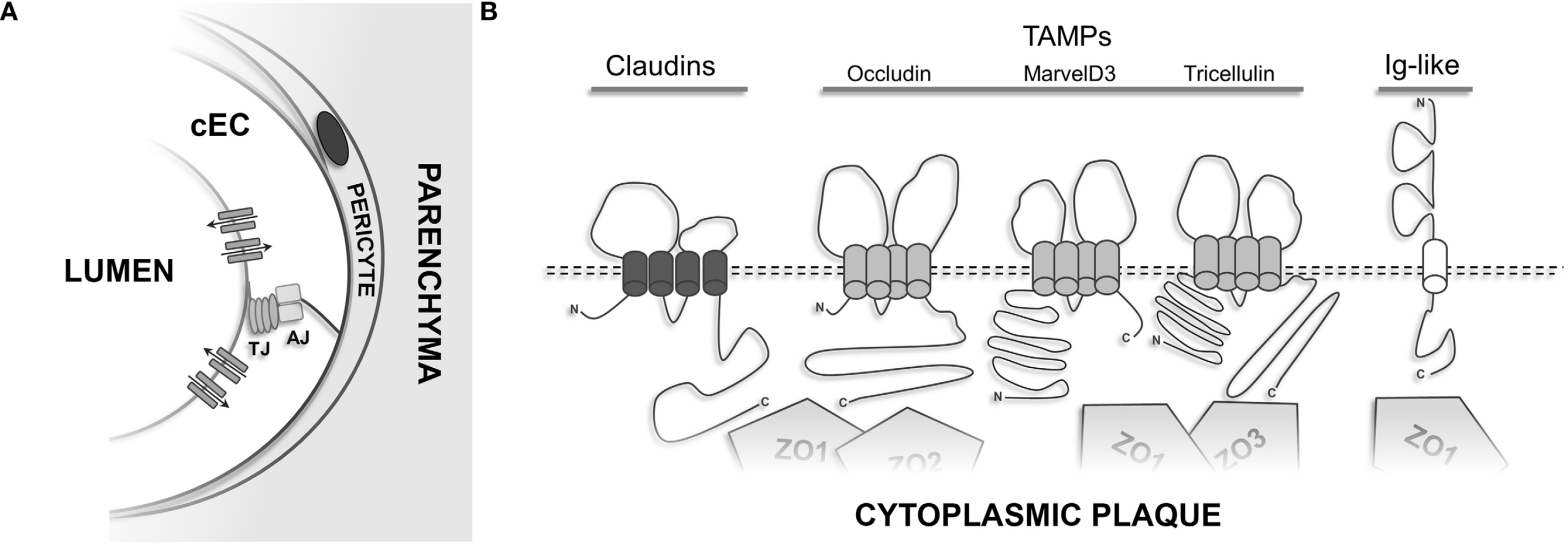
**Transmembrane proteins of the TJ. (A)** Cerebral endothelial cells constitute the cellular building block of the BBB and control the para- and transcellular routes via elaborate influx and efflux transporters and cell-cell adhesion structures. Astroglial endfeet, neurons, and microglia which are present at the neurovascular unit have been omitted for clarity. **(B)** Schematic membrane-spanning models of the transmembrane components present at TJs.

Overall, 3 distinct TJ-associated transmembrane protein groups can be distinguished (see Figure 4B): (1) claudins, a family of proteins comprising at least 27 different members in mammals (Mineta et al., 2011); (2) TAMPs (TJ-associated MARVEL proteins)—a group of proteins containing the structural tetraspanning MARVEL motif, including occludin (Furuse et al., 1993), tricellulin (Ikenouchi et al., 2005), and MarvelD3 (Steed et al., 2009); (3) Immunoglobulin superfamily membrane proteins JAM-A/-B/-C (Martin-Padura et al., 1998; Aurrand-Lions et al., 2000; Palmeri et al., 2000; Ebnet et al., 2003), coxsackie adenovirus receptor (CAR) (Carson et al., 1999; Cohen et al., 2001), and endothelial cell-selective adhesion molecule (ESAM) (Nasdala et al., 2002).

*Occludin* was the first integral membrane protein shown to localize to TJs (Furuse et al., 1993) and is characterized by four transmembrane domains, a short intracellular turn, two extracellular loops, and a long carboxy- and a short amino-terminal cytoplasmic domain. Cis-oligomerization of occludin seems to be mediated by the MARVEL motif (Blasig et al., 2006; Yaffe et al., 2012), whereas the extracellular loops are believed to contribute to the gate-keeping function of TJs. The last 150 aa of the carboxyl terminus of occludin interact directly with F-actin (Wittchen et al., 1999) and the scaffold proteins ZO-1 (Furuse et al., 1994), ZO-2 (Itoh et al., 1999b), and ZO-3 (Haskins et al., 1998). Information regarding the physiological relevance of the N-terminal domain remains scarce. Using a yeast-based two-hybrid screen the E3 ubiquitin protein ligase Itch was found to directly interact with the outermost NH_2_-terminal domain of occludin (Traweger et al., 2002). Indeed, following VEGF-induced phosphorylation occludin is targeted for proteasomal degradation via Itch, resulting in increased endothelial permeability (Murakami et al., 2009). Occludin is targeted by several kinases and is phosphorylated on serine, threonine (Sakakibara et al., 1997; Wong, 1997), and tyrosine residues (Tsukamoto and Nigam, 1999; Chen et al., 2002) and reversible phosphorylation is an important regulatory mechanism to control TJ assembly and maintenance. For example, endothelial cells subjected to shear stress showed a significantly reduced, but tyrosine-phosphorylated pool of occludin (DeMaio et al., 2001). However, controversial results on the role of occludin phosphorylation in epithelial and endothelial cells exist.

Early investigations led to the assumption that occludin is the core transmembrane protein of TJs (Furuse et al., 1996; McCarthy et al., 1996). However, the TJ scientific community was stunned to learn that occludin is dispensable for the formation of tight junction strands. Occludin-deficient ES cells were found to be morphologically indistinguishable from their wild type counterparts (Saitou et al., 1998) and occludin-deficient mouse embryos were viable and did not show any gross morphological alterations of TJs (Saitou et al., 2000). However, phenotypically *ocln*-/- animals displayed multiple histological abnormalities, including chronic inflammation and hyperplasia of the gastric epithelium, calcification of the brain, cortical bone thinning, and testicular atrophy. Subsequent analysis of the knockout animals suggested occludin may play a role in epithelial differentiation and proliferation (Schulzke et al., 2005). In man, mutations of the occludin gene cause band-like calcification with simplified gyration and polymicrogyria (BLC-PMG), a rare neurological disorder resulting in malformations of cortical development, implicating a role of occludin for TJs at the BBB (O'Driscoll et al., 2010). Most likely, other proteins of the TAMP family (i.e., tricellulin or MarvelD3) act redundantly partially masking the function of occludin. In summary, although occludin clearly is not of fundamental importance for the core TJ structure the findings outlined above suggest that it has a regulatory and accessory function in TJ formation and physiology.

After having realized that occludin is not the major TJ-strand protein, the search for a new protein began. Furuse and Tsukita identified small proteins that co-fractionated with occludin in a junction-enriched chicken liver fraction (Furuse et al., 1998). Since the initial discovery of these small proteins, termed claudin-1 and claudin-2, a total of 27 different *claudin* family members have been identified in man and mouse (Mineta et al., 2011). Although the predicted protein folding topology of claudins (21–28 kD) is very similar to that of occludin, they do not share any sequence homology (Furuse et al., 1998). Several gain-of-function and loss-of-function studies have led to the current model that claudins are the prime transmembrane proteins responsible for the formation of higher-order tight junction strands. For example, claudin-1 deficient mice die shortly after birth due to excessive transepidermal water loss (Furuse et al., 2002) and claudin-11 knockout animals show a complete loss of TJs in Sertoli cells, oligodendrocytes, and the marginal cells of the inner ear (Gow et al., 1999, 2004; Mazaud-Guittot et al., 2010).

It is believed that claudins and TAMPs oligomerize in a homo- and heterophilic fashion and TJ-strands are formed by mosaics of different claudin-family members and the combination and stoichiometry of various isoforms determines the charge- and size-selective properties of the barrier (Sonoda et al., 1999; Anderson and Van Itallie, 2009). Further, claudins show a tissue-specific expression pattern, contributing to tissue specific barrier properties. Therefore, for each tissue a unique combination of claudins which oligomerize with other TAMPS most likely determines the paracellular tightness of the junction toward different solutes.

Overall, claudins-1, -3, -5, -11, -14, -19 appear to be sealing components of tight junctions and for several of them human mutations have been characterized. Mutations in claudin-1 cause the neonatal ichtyosis and sclerosing cholangitis (NISCH) syndrome. Claudin-14 mutations are the underlying cause of autosomal recessive deafness 29 and 49 and mutations in claudin-19 cause renal hypomagnesemia with ocular involvement. Next to acting as “sealants” claudins have been proposed to form paracellular ion-selective channels (Amasheh et al., 2002) and so far claudin-2, -10a, -10b, -15, and -17 have been shown to have channel-forming properties. Whereas claudins-2, 10b, and 15 display cation selectivity, -10a and 17 have been described as anion-selective channels (reviewed in Gunzel and Fromm, 2012).

At the BBB, mainly claudin-3 (Wolburg et al., 2003) and claudin-5 (Morita et al., 1999) are being expressed, claudin-12 likely also being part of the BBB-TJ (Ohtsuki et al., 2007; Schrade et al., 2012). Early studies also have demonstrated the presence of claudin-1 at brain capillary endothelial cells (Liebner et al., 2000a), however this was a false report due to antibody cross-reactivity with claudin-3. Subsequent studies using claudin-1 specific antibodies failed to detect any claudin-1 in CNS parenchymal microvessels and/or in primary mouse or human brain endothelial cells (Wolburg et al., 2003; Hamm et al., 2004; Coisne et al., 2005; Weksler et al., 2005).

Of those having been described so far, claudin-5 is the most abundant isoform at the BBB as also shown by a SAGE analysis of rat brain microvasculature (Enerson and Drewes, 2006). It is crucial for the “tightness” of TJs at the mammalian BBB as its deletion resulted in a size-selective opening of the BBB *in vivo*, allowing the diffusion of molecules smaller than 800 Da (Nitta et al., 2003). Interestingly, the TJs of the BBB were morphologically normal, nevertheless the knockout of claudin-5 resulted in early neonatal death. This study also shows that the increase in paracellular flux is tolerated during embryogenesis, potentially due to the presence of the placental barrier. This indicates that the BBB in the mouse embryo is “immature,” even though it already restricts the flux of blood-borne products into the neuroparenchyma (see Figures 1C,D).

Additional data establishes the key role of claudin-5 and claudin-3 in TJ integrity at the BBB. For example, overexpression of claudin-5 increases the paracellular tightness in cultured brain microvascular endothelial cells (Ohtsuki et al., 2007) and the selective loss of claudin-3 in experimental autoimmune encephalomyelitis or human glioblastoma multiforme is associated with BBB breakdown, suggesting that it also contributes to BBB integrity and function (Wolburg et al., 2003). Interestingly, a specific role of claudin-3 at the BBB is also underscored by the observation that its expression correlates with the maturation of the BBB in response to Wnt/β-catenin signaling during development (Liebner et al., 2008). However, the exact role of claudin-3 at the BBB remains to be seen.

The greatest sequence diversity of claudins is located in the C-terminal domain, suggesting that their diverse functions are in part conferred by their association with cytoplasmic proteins. Generally, the last C-terminal amino acids constitute a PDZ binding motif resulting in the recruitment of the TJ-plaque proteins ZO-1, ZO-2, ZO-3 (Itoh et al., 1999a), which seem to instruct claudins to form functional TJ strands (Umeda et al., 2006). Similarly to occludin, claudins are also phosphorylated on serine or threonine residues, regulating their function and intracellular localization (Ishizaki et al., 2003; D'Souza et al., 2005; Ikari et al., 2006; Aono and Hirai, 2008).

Next to bicellular contacts, the formation of specialized tricellular contacts is required to maintain the integrity of cellular sheets. At these contacts a specialized TJ structure (tTJs) is formed where three sealing elements are in close apposition and form a narrow tube in the intercellular cleft (Friend and Gilula, 1972; Staehelin, 1973; Ikenouchi et al., 2005). Specialized transmembrane proteins, tricellulin and proteins of the angulin family are located at these contacts and recently have been identified in endothelial cells of the brain and retina (Mariano et al., 2013; Iwamoto et al., 2014). Tricellulin shares some sequence similarity with occludin and depending on the cell type can also be found at bicellular contacts where it possibly acts as a substitute for occludin (Ikenouchi et al., 2005; Krug et al., 2009). Taken together, TJ-associated MARVEL proteins are able to partially exert redundant functions, but also show tissue-specific expression and are involved in distinct aspects of tight junction assembly, maintenance, and regulation (Raleigh et al., 2010).

So far, the last class of transmembrane proteins found to localize to the TJ are members of the immunoglobulin superfamily. *Junction adhesion molecules* or more precisely the first isoform JAM-A was identified by using monoclonal antibodies raised against endothelial antigens (Martin-Padura et al., 1998). They form homotypic cell-cell contacts between endothelial and epithelial cells and are highly enriched at TJs (Bazzoni et al., 2000a; Liu et al., 2000). JAMs are generally glycosilated and encompass two extracellular variable type Ig domains and a single transmembrane domain. Through a classical type II PDZ binding motif located in its outermost C-terminus JAM-A interacts with several cytoplasmic proteins, aiding in the establishment of the cytoplasmic plaque of TJs (Bazzoni et al., 2000b; Ebnet et al., 2000; Martinez-Estrada et al., 2001; Hamazaki et al., 2002). Interestingly, JAM-/- mice show little alterations of the overall epithelial architecture in the intestinal tract, but display increased colonic inflammation and paracellular permeability (Laukoetter et al., 2007). Next to JAM-A, the closely related proteins JAM-B (JAM2) and JAM-C (JAM3) have been cloned and characterized (Aurrand-Lions et al., 2000; Cunningham et al., 2000; Palmeri et al., 2000; Arrate et al., 2001; Aurrand-Lions et al., 2001). In contrast to JAM-A, JAM-B/-C seem restricted to endothelial cells and have been demonstrated to form heterodimers in cell-cell contacts, thereby counteracting the interaction with the leukocyte receptor alpha(M)beta2 integrin (Cunningham et al., 2002; Lamagna et al., 2005). In humans, homozygous mutations of JAM-C result in intracerebral hemorrhages, subependymal calcification, and congenital cataracts (Mochida et al., 2010). However, overall little is known about their exact role at the BBB.

Strikingly, among all TJ-associated transmembrane proteins only JAM-A/-B/-C interact directly with the cell polarity protein Par3 (Ebnet et al., 2001, 2003; Itoh et al., 2001). JAMs localize to sites of early cell-cell contact formation, so called puncta or primordial spot-like junctions, where they most likely “flag” this region for the recruitment of the Par3/Par6/aPKC polarity complex during early junctional biogenesis in epithelial and endothelial cells (see also further above). Next to their involvement in cell-cell adhesion JAMs also mediate the transendothelial migration of leukocytes which is of particular importance for BBB function (Fraemohs et al., 2004; Aurrand-Lions et al., 2005).

Four additional Ig-superfamily protein members have been found to localize to TJs: CAR—coxsackie- and adenovirus receptor (Cohen et al., 2001; Raschperger et al., 2006), CLMP-coxsackie- and adenovirus receptor-like membrane protein (Raschperger et al., 2004), JAM 4 (Hirabayashi et al., 2003), and ESAM—endothelial cell-selective adhesion molecule (Hirata et al., 2001; Nasdala et al., 2002). Of these, ESAM seems to be the only endothelial-specific transmembrane protein. ESAM-/- mice did not show any major vascular defects, however endothelial tube formation was impaired suggesting a role of ESAM in cell-cell contact formation (Ishida et al., 2003). More recently, ESAM was also suggested to support the extravasation of neutrophils during the early phase of an inflammatory response (Wegmann et al., 2006).

Taken together, transmembrane proteins of the TJ not only facilitate cell-cell adhesion and impede paracellular flux, they also target cytoplasmic protein assemblies to distinct cell membrane domains, endowing endothelial and epithelial cells with their critical biological properties.

### TJ cytoplasmic plaque proteins

**Figure 5:**
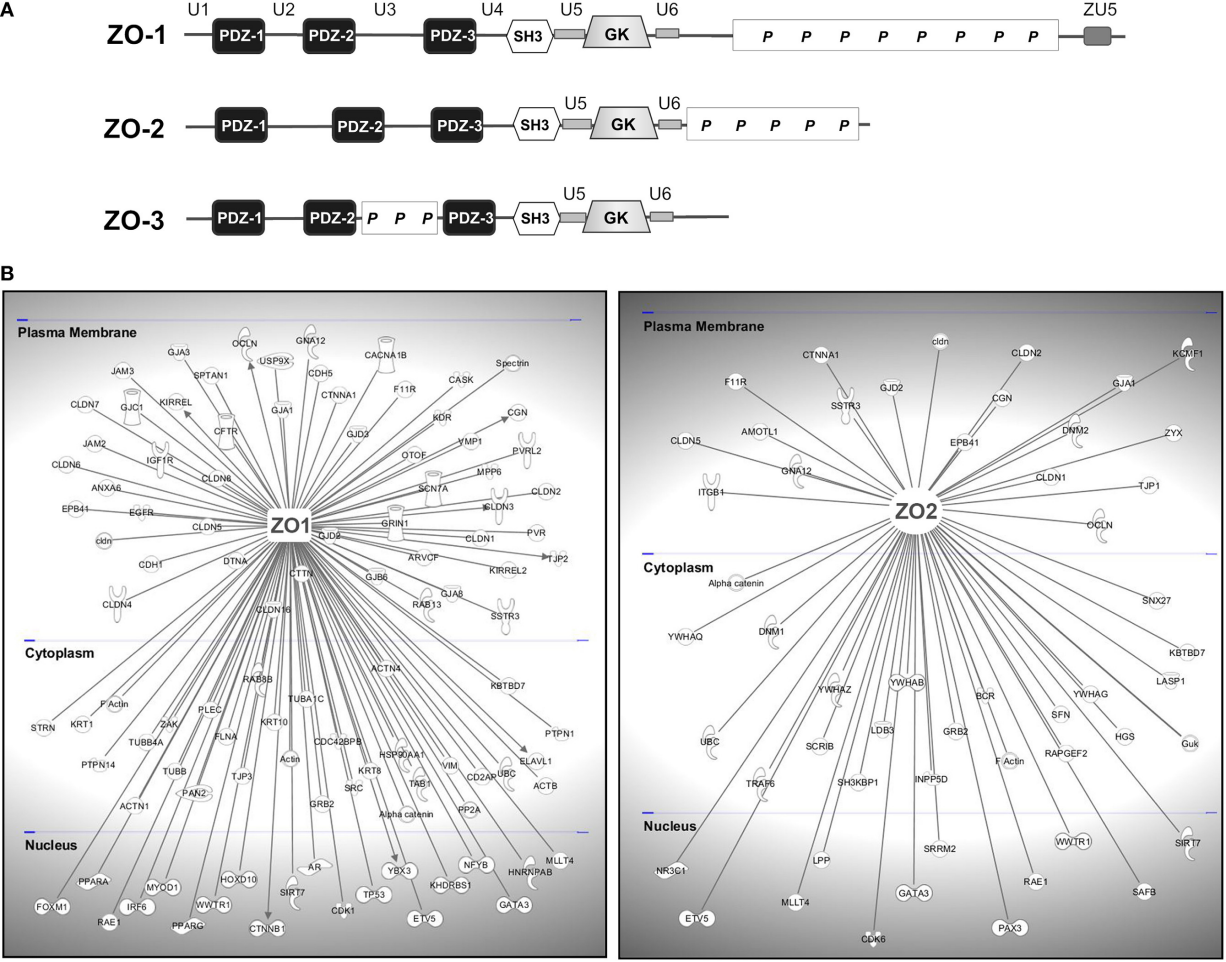
**TJ-MAGUKS are membrane-associated adaptor proteins assembling the tight junction plaque. (A)** ZO-1, ZO-2, and ZO-3 are multi-domain proteins composed of three PDZ domains, one SH3 and a catalytically inactive GUK domain. Next to these MAGUK-related domains, ZO proteins also contain various ZO-specific unique (U) and proline-rich (*P*) regions. ZU5 is a region found in ZO-1 and Unc5-like netrin receptors. **(B)** ZO-1 and ZO-2 are at the basis of large protein assemblies containing structural and signaling molecules. Shown are proteins that have been demonstrated to interact with ZO-1 or ZO-2 in epithelial and endothelial cells. Gene symbols according to the H.G.N.C. nomenclature are indicated.

The most prominent subgroup of scaffolding proteins localizing to tight junctions is represented by the MAGUK (membrane-associated guanylate kinase) proteins (Dimitratos et al., 1999; Gonzalez-Mariscal et al., 2000; Funke et al., 2005). The modular nature of MAGUK proteins was early recognized as they all share a common structural core consisting of one or several PDZ domains and an SH3 domain followed by a catalytically inactive guanylate kinase (GUK) domain (see Figure 5A). Originally, MAGUKs only referred to a group of proteins including the mammalian synaptic scaffold protein Psd-95, the Drosophila tumor suppressor Dlg, and the tight junction protein ZO-1. Since then a large number of scaffold proteins belonging to this family have been identified. Generally, at junctions they serve as molecular hubs coordinating large protein assemblies which transduce the signals impinging on and emanating from the apical plasma membrane, thereby influencing diverse cellular processes, including the establishment and maintenance of cell polarity and cell-cell adhesion complexes, synaptic plasticity, and cell survival (Funke et al., 2005; Te Velthuis et al., 2007; De Mendoza et al., 2010).

Stevenson and Goodenough identified the first TJ-associated MAGUK ZO-1 (zonula occludens-1) by generating monoclonal antibodies against a TJ-enriched plasma membrane fraction from rodent liver (Stevenson et al., 1986). Subsequently, ZO-2 (Gumbiner et al., 1991) and ZO-3 (Balda et al., 1993; Haskins et al., 1998) were discovered by co-immunoprecipitation studies. The ZO proteins are at the core of large protein networks which are anchored to the TJ through their association with the C-terminal domains of JAM-A (Bazzoni et al., 2000b; Ebnet et al., 2000; Itoh et al., 2001), occludin (Furuse et al., 1994; Fanning et al., 1998), and most claudins (Itoh et al., 1999a). They further interact physically or functionally with a multitude of cytoplasmic proteins forming the TJ-plaque and they provide a direct link to the actin cytoskeleton via a direct interaction with actin filaments (Itoh et al., 1997; Fanning et al., 1998; Wittchen et al., 1999; Fanning et al., 2002) or the actin binding proteins fodrin/spectrin, cortactin, and protein 4.1R (Katsube et al., 1998; Mattagajasingh et al., 2000). This association to intact cytoskeletal arrays is critical for the physiology of cell–cell junctions.

ZO-1 and ZO-2 are expressed in both epithelial and endothelial cells and have been shown to be indispensable for TJ- strand formation (Umeda et al., 2006; Katsuno et al., 2008). ZO-3 seems to be confined to epithelial cells (Inoko et al., 2003) and is less critical for TJ function (Adachi et al., 2006; Xu et al., 2008). Structurally, ZO proteins share a high degree of sequence similarity across their protein-binding modules, however display significant variability within their C-terminus suggesting that these regions are functionally distinct. Next to the core MAGUK domains of the ZO proteins, several unique (U) regions have been identified (see Figure 5A). In the case of ZO-1 these regions influence its intracellular localization and its scaffolding properties (Fanning et al., 2007; Fanning and Anderson, 2009), but little is known about their relevance for ZO-2 and ZO-3. Although the high sequence similarity also suggests a fair degree of functional redundancy, specific roles have been described for the different ZO proteins. Deficiency of ZO-1 results in mislocalization of endothelial junctional adhesion complexes and defects in angiogenesis were evident, resulting in early embryonic lethality (Katsuno et al., 2008). Deletion of ZO-2 in mice is embryonic lethal shortly after implantation due to an arrest in early gastrulation, embryos showing a decreased proliferation rate and an increase in apoptotic cells (Xu et al., 2008).

In brain cECs a decrease in the expression of ZO-1 and occludin after cerebral embolism has been reported (Kago et al., 2006) and *in vitro* under hypoxic conditions the localization of ZO-1 and ZO-2 was altered with a concurrent decrease in TEER (Mark and Davis, 2002; Fischer et al., 2004). Further, disruption of the TJs at the BBB occurs in many neurological disorders (Rosenberg, 2012). However, in most cases the changes in TJ structure and function are most likely the result of indirect downstream signaling events triggered secondary to an inflammatory or traumatic event and are not directly exerted by the junctional proteins themselves. Generally, the reduced expression of ZO-1 correlates with increased proliferation of cells and/or transformation. For example, in highly proliferative brain microvascular endothelial cells from human brain tumors ZO-1 levels are low. Apart from reduced expression, ZO proteins may also regulate proliferation by nuclear translocation. A large body of evidence demonstrates that ZO-1 and ZO-2 are not only present at cell-cell junctions but also target the cell nucleus. Even though the majority of these findings relate to epithelial cells, it is likely that many of these processes also take place in endothelial cells. All ZO proteins harbor conserved nuclear localization and nuclear export sequences (Lopez-Bayghen et al., 2006) and in sparse cultures, ZO-1 and ZO-2 are present in cell nuclei but become re-distributed to the plasma membrane as soon as cells reach confluence (Gottardi et al., 1996; Islas et al., 2002; Traweger et al., 2003; Jaramillo et al., 2004; Gonzalez-Mariscal et al., 2006). ZO-1 further perturbs gene expression by sequestering the Y-box transcription factor ZONAB to the cytoplasm. ZONAB interacts with the cell cycle regulator CDK4 and controls expression of cell cycle regulators such as cyclin D1 and PCNA (for review see Balda and Matter, 2009). ZO-2 has been shown to inhibit the transcription of G1 cyclin after translocating to the nucleus (Gonzalez-Mariscal et al., 2009). In addition, several proteins interact with ZO-2 during nuclear shuttling or within the nucleus itself. For example, the interaction of the armadillo-repeat protein ARVCF (armadillo repeat gene deleted in velocardiofacial syndrome) depends on an N-terminal PDZ domain of ZO-2 (Kausalya et al., 2004), a region which is also targeted by the non-receptor tyrosine kinase JAK1. Also, the transcription factors Jun, Fos, and C/EBP were shown to associate with ZO-2 within the nucleus and at TJs in epithelial cells (Betanzos et al., 2004). Next to directly associating with and/or influencing transcription factors, ZO-2 possibly impacts upon the transcription machinery via its association with the hnRNP SAF-B/HET (Traweger et al., 2003), a molecular platform which assembles a transcription complex in the vicinity of actively transcribed genes (Renz and Fackelmayer, 1996). Taken together, ZO proteins potentially act as direct or indirect regulators of cell proliferation through nuclear translocation and/or their ability to sequester transcription factors and signaling molecules to the cytoplasm. However, our understanding of the signaling pathways and their biochemical properties through which ZO proteins exert their effects beyond cell-cell adhesion remains fragmentary.

Next to the TJ-MAGUKs members of the so called MAGI protein family (membrane-associated guanylate kinase with inverted orientation) localize to TJs. They are large adaptor proteins containing 6 PDZ domains and a module of a GuK domain and two WW domains C-terminal to the first PDZ domain. Of the identified MAGI proteins, only MAGI-1 has been shown to localize to TJs of brain endothelial cells where it interacts with ESAM and promotes maturation of TJs in a Rho-dependent manner (Wegmann et al., 2004; Kimura et al., 2010). In vascular endothelial cells MAGI-1 physically interacts with β-catenin and localizes to VE-cadherin-based AJs (Sakurai et al., 2006), however much detail about the role of MAGI-1 at AJs and TJs is still missing.

Several other regulatory cytoplasmic plaque proteins have been demonstrated to localize to the TJ-plaque, including heterotrimeric G-proteins (Denker et al., 1996; Fabian et al., 1998; Saha et al., 1998). In brain capillary endothelial cells G_αi2_ is found in a complex together with claudin-5 and its depletion results in increased paracellular permeability and delayed TJ-formation after a hyperosmotic shock of cultured cECs (Luissint et al., 2012). Another class of proteins influencing TJ physiology are Rho family GTPases. In rat brain endothelial cells C3 transferase, a toxin which specifically inactivates RhoA-C, abrogated lymphocyte transmigration, suggesting that Rho is required for junction intergrity (Adamson et al., 1999). In addition, expression of mutant Rho GTPases in epithelial and endothelial cells affected barrier properties (Nusrat et al., 1995; Jou et al., 1998; Wojciak-Stothard et al., 2001; Bruewer et al., 2004) and the paracellular flux of sucrose was increased in cerebral endothelial cells treated with lysophoshatidic acid (Schulze et al., 1997). So far, 3 TJ-associated Rho-specific regulators have been identified. (1) The Rho-activator GEF-H1 has been shown to regulate paracellular permeability in epithelial and endothelial cells (Benais-Pont et al., 2003; Birukova et al., 2006). (2) Tuba, an activator for Cdc42, which is recruited to epithelial TJs in a ZO-1-dependent manner (Otani et al., 2006). (3) The GAP protein SH3BP1 which restricts the activity of Cdc42 and Rac when complexed with the scaffold protein CD2AP, parcingulin, and the actin filament capping protein CapZ (Elbediwy et al., 2012).

Cingulin and the closely related protein paracingulin (JACOP) have also been shown to concentrate at TJs of epithelial and endothelial cells, providing a direct link to the cytoskeleton and to regulate the activity of Rho family GTPases at junctions by recruiting guanidine exchange factors of RhoA and Rac1 (Paschoud et al., 2011). Overall, little is known about how the activities of RhoGTPases are regulated and how they feed into the signaling networks that control tight junction formation and integrity. Most likely a Rho-dependent regulation of the actinomyosin cytoskeleton perturbs TJ integrity.

Taken together, the modular structure of a set of adaptor proteins allows the spatio-temporal assembly of multi-protein complexes (see Figure 5B) at discrete regions of epithelial and endothelial cells through their interaction with clustered transmembrane TJ proteins, not only ensuring the structural integrity of the TJ, but also integrating various regulatory pathways at the cytoplasmic plaque which are pivotal for TJ physiology.

## The NVU—coupling BBB integrity and neuronal activity

The endothelial cells forming the lining of the microvasculature of the brain are set up to actively provide the brain with oxygen and essential nutrients, allowing not only a timely response to local demands but also buffering, which is crucial to physiological neuronal function (Abbott et al., 2006; Abbott, 2013). Brain cECs are wrapped by pericytes, both sharing a common basement membrane onto which astrocytes project their endfeet, ensheathing over 95% of the abluminal microvascular surface (see Figure 4A). In addition, cECs receive input from neurons.

Early *in vivo* grafting studies and experiments using astrocyte-conditioned media highlighted the role of astrocyte-derived secreted factors to influence the BBB properties of brain cECs by inducing junction formation (Arthur et al., 1987; Janzer and Raff, 1987; Tao-Cheng et al., 1987; Haseloff et al., 2005). Glial-derived neurotrophic factor (GDNF) has been demonstrated to enhance the barrier properties of brain cECs *in vitro* (Igarashi et al., 1999) and FGF was found to decrease BBB permeability (El Hafny et al., 1996), which is in line with the BBB integrity loss seen in FGF-2^−/−^/FGF-5^−/−^ double mutant mice (Reuss et al., 2003). Recently, sonic hedgehog (Shh) has been shown to be secreted by perivascular astrocytes and endothelial-specific deletion of Smoothened (Smo), a downstream target of Shh, resulted in incomplete BBB maturation early during mouse embryonic development (Alvarez et al., 2011). Further, Shh knockout mice displayed a decrease in TJ proteins in brain cECs.

Next to astrocytes, pericytes have been shown to directly influence the BBB (Hori et al., 2004; Daneman et al., 2010b). The canonical Wnt/β-catenin has also been shown to influence TJ integrity and BBB formation, increasing the expression of several genes, including the TJ protein claudin-3 (Liebner et al., 2008; Daneman et al., 2009). *In vitro*, activation of Wnt pathway controls the expression several TJ proteins (Paolinelli et al., 2013). Further, genetic ablation of Wnt7a/b results in the breakdown of the BBB and a severe CNS-specific hemorrhaging phenotype early in embryonic development (Stenman et al., 2008).

Next to astrocytes and pericytes, noradrenergic, serotonergic, cholinergic, and GABA-ergic neurons have been found to directly contact brain cECs (Cohen et al., 1996, 1997; Tong and Hamel, 1999; Vaucher et al., 2000). It is believed that neurons innervating the brain cECs and astrocytes of the NVU regulate BBB permeability; however the mechanism of action remains unknown.

Taken together, the BBB is a dynamic structure receiving continuous input from the CNS. It is this intimate association of cerebral endothelial cells with neuroectoderm-derived accessory cells that influences barrier function (i.e., tightness) in a spatio-temporal manner in the developing and mature brain.

## Concluding remarks

The basic structure of the BBB has been described more than 100 years ago. Much progress has been made on elucidating the molecular programs that govern tight junction biogenesis and many of the core molecular components of the endothelial TJ complex which constitutes the main physical paracellular barrier at the BBB have been identified. TJs are now appreciated as dynamic structures where multiple signaling pathways converge, fitting the BBB of the cerebral vascular bed with a high degree of plasticity in response to physiological and pathological stimuli. Next to cell-cell adhesion, processes such as cellular polarization, cytoskeletal rearrangements, integrin-mediated attachment to the ECM, and polarized trafficking are pivotal to the physiological functioning of the BBB. These mechanisms have so far mainly been described in epithelial cells and although most of the key players are present in endothelial cells, their actions may vary markedly in different physiological contexts. Therefore, it will be central to gain a better understanding of the spatio-temporal establishment of functional TJs at the BBB during early vascularization of the embryonic brain. Clearly, further insights have largely been hampered by the lack of suitable *in vitro* culture systems appropriately replicating the vascular properties of the brain microcapillaries. As for many neurological diseases it remains problematic to deliver appropriate drugs to the CNS, a transient modulation of the TJs or transcytosis at the BBB may constitute an alternative approach. Undoubtedly, a more detailed understanding of the complex structure and physiology of endothelial TJs will aid in developing practical solutions for the future.

### Conflict of interest statement

The authors declare that the research was conducted in the absence of any commercial or financial relationships that could be construed as a potential conflict of interest.

## Abbreviations

CD31/34: cluster of differentiation 31/34
Cdc42: cell division control protein 42
CD2AP: CD2-associated protein
CDK4: cyclin-dependent kinase 4/cell division protein kinase 4
C/EBP: CCAAT/enhancer-binding-protein
cECs: capillary endothelial cells
CNS: central nervous system
ECM: extracellular matrix
ES cells: embryonic stem cells
GAP: GTPase activating protein
GDNF: glial-derived neurotrophic factor
GEF-H1: guanine nucleotide exchange factor-H1
Glut-1: glucose transporter-1
GUK: guanylate kinase
hCMEC/D3: human cerebral microvascular endothelial cell line/D3
hnRNP: heterogeneous nuclear ribonucleoprotein
MAGI: membrane-associated guanylate kinase inverted
MAGUK: membrane-associated guanylate kinase
MARVEL: MAL (myelin and lymphocyte) and related proteins for vesicle trafficking and membrane link
NVU: neurovascular unit
PDZ: PSD-95, discs large, ZO-1
SMAD: SMA (small)/ MAD: Mothers against decapentaplegic
TAMPs: TJ-associated MARVEL proteins
TEER: transepithelial electrical resistance
Unc-5: uncoordinated locomotion-5
VEGF: vascular endothelial growth factor
WD40: tryptophan-aspartic acid (WD) repeat
Wnt: wingless/Int-1.
